# The role IL-1 in tumor-mediated angiogenesis

**DOI:** 10.3389/fphys.2014.00114

**Published:** 2014-03-28

**Authors:** Elena Voronov, Yaron Carmi, Ron N. Apte

**Affiliations:** The Shraga Segal Department of Microbiology, Immunology and Genetics, Faculty of Health Sciences and The Cancer Research Center, Ben-Gurion University of the NegevBeer-Sheva, Israel

**Keywords:** IL-1α, IL-1β, VEGF, VEGFR1, VEGFR2, angiogenesis, myeloid cells, inflammation

## Abstract

Tumor angiogenesis is one of the hallmarks of tumor progression and is essential for invasiveness and metastasis. Myeloid inflammatory cells, such as immature myeloid precursor cells, also termed myeloid-derived suppressor cells (MDSCs), neutrophils, and monocytes/macrophages, are recruited to the tumor microenvironment by factors released by the malignant cells that are subsequently “educated” *in situ* to acquire a pro-invasive, pro-angiogenic, and immunosuppressive phenotype. The proximity of myeloid cells to endothelial cells (ECs) lining blood vessels suggests that they play an important role in the angiogenic response, possibly by secreting a network of cytokines/chemokines and inflammatory mediators, as well as via activation of ECs for proliferation and secretion of pro-angiogenic factors. Interleukin-1 (IL-1) is an “alarm,” upstream, pro-inflammatory cytokine that is generated primarily by myeloid cells. IL-1 initiates and propagates inflammation, mainly by inducing a local cytokine network and enhancing inflammatory cell infiltration to affected sites and by augmenting adhesion molecule expression on ECs and leukocytes. Pro-inflammatory mediators were recently shown to play an important role in tumor-mediated angiogenesis and blocking their function may suppress tumor progression. In this review, we summarize the interactions between IL-1 and other pro-angiogenic factors during normal and pathological conditions. In addition, the feasibility of IL-1 neutralization approaches for anti-cancer therapy is discussed.

## Introduction

Postnatal angiogenesis, first described by Folkman in the 70's, referred to a process by which mature endothelial cells (ECs) proliferate and sprout to create new blood vessels. Usually, quiescent ECs are inactive for months and even years; they proliferate only upon angiogenic activation due to various pathological conditions (Carmeliet and Jain, 2000; Carmeliet, 2003, 2005; Ribatti, 2009). Initially, the angiogenic process was considered to represent the outcome of straightforward interactions between pro-angiogenic factors and specific signaling receptors on the membranes of ECs (Folkman, 1995, 2001, 2003; Folkman and D'Amore, 1996). Although many molecules are involved in angiogenesis, VEGF stands out because it has a direct mitogenic effect on ECs and functions as a key regulator of both physiological and pathological angiogenesis (Thomas, 1996; Alitalo and Carmeliet, 2002; Shibuya, 2006a,b). VEGFA belongs to the VEGF family and through activation of two tyrosine kinase receptors, VEGFR1 (Flt-1) and VEGFR2 (FLK-1), plays a major role in the angiogenic response. The biological effects of VEGF are thought to be mediated mainly by VEGFR2, whereas VEGFR1 can transduce a weak, intracellular signal in ECs and was initially thought to be a decoy receptor, thus serving as a negative regulator of angiogenesis (Hiratsuka et al., 1998). At the same time, VEGFR1 is expressed on different myeloid cells, such as macrophages and is directly involved in the migration of these cells to inflammatory areas (Shibuya, 2006a,b). Recently, it was found that inflammation usually accompanies tumor development and the mechanism by which inflammation contributes to tumor-mediated angiogenesis has been the subject of intensive study.

Pro-inflammatory cytokines are potent factors in the induction and support of angiogenesis. Of special relevance to angiogenesis are the “alarm cytokines” IL-1 and TNFα that are generated by macrophages immediately after confrontation with inflammatory stimuli. These stimuli induce the expression of pro-inflammatory genes in diverse stromal/inflammatory cells, which ultimately results in a local cascade of cytokines and small effector molecules that initiate, propagate, and sustain inflammation (Dinarello, 1996; Apte and Voronov, 2002, 2008; Balkwill, 2002; Apte et al., 2006a,b; Balkwill and Mantovani, 2012). IL-1 and TNFα also increase the expression of adhesion molecules on both ECs and leukocytes, promoting leukocyte infiltration from the blood into inflamed tissues (Apte and Voronov, 2002, 2008; Apte et al., 2006a,b).

The direct relationship between inflammation and tumor-mediated angiogenesis has prompted the development of novel anti-tumor therapies based on the attenuation of inflammation.

In this review, the interaction of IL-1 molecules with a critical pro-angiogenic factor, VEGF, and the effects of IL-1 on ECs and myeloid cells during the angiogenic response are discussed.

## The IL-1 molecules

The IL-1 family consists of agonistic and antagonistic molecules, as well as receptors. The two major agonistic proteins are IL-1α and IL-1β. The third important molecule is the IL-1 receptor antagonist (IL-1Ra), which is a physiological inhibitor of pre-formed IL-1; it binds to IL-1 receptors without transmitting an activation signal (reviewed in Dinarello, 1996; Stylianou and Saklatvala, 1998; Sims et al., 2001; Apte and Voronov, 2002, 2008; Arend, 2002; Dinarello, 2002; O'Neill, 2002; Braddock and Quinn, 2004; Apte et al., 2006a,b; Mantovani et al., 2008; Mantovani and Sica, 2010).

Many cell types produce and secrete IL-1α, IL-1β, and IL-1Ra upon activation with microbes, microbial products, cytokines, and other environmental stimuli, as well as products of damaged tissue (reviewed in Dinarello, 1996, 2006; Apte and Voronov, 2008; Voronov et al., 2013). IL-1α and IL-1β are synthesized as precursors of 31 kD that are further processed by proteases to their mature secreted 17 kD forms. IL-1 differs from most other cytokines by the lack of a signal sequence, thus not passing through the endoplasmic reticulum-Golgi pathway. The mechanisms of IL-1 secretion are not yet completely understood. IL-1Ra, which has a signal peptide, is secreted in the ER-Golgi exocytic pathway.

IL-1β is not present in homeostatic conditions; it is induced and secreted only upon inflammatory signals. The IL-1β precursor is biologically inactive until it is enzymatically cleaved into the mature secreted form by the IL-1β-converting enzyme (caspase-1) that is activated in the cytosol on the inflammasome platform (reviewed in Dinarello, 2009, 2011a; Eisenbarth and Flavell, 2009; Franchi et al., 2009; Martinon et al., 2009; Latz, 2010; Schroder and Tschopp, 2010). IL-1β, due to the fact that it is extensively secreted, has been considered to be the major pro-inflammatory molecule. At low local doses, IL-1β induces limited inflammatory responses, followed by activation of specific immune mechanisms, while at high doses, IL-1β induces broad inflammation accompanied by tissue damage and immune suppression (reviewed in Voronov et al., 2013).

IL-1α, in contrast, is present in homeostatic conditions in many cells. IL-1α is only rarely secreted by living cells and in most cases is undetectable in body fluids. Its protein is translated as a precursor (ProIL-1α), which is further processed by the Ca^2+^-dependent protease, calpain, into the mature 17 kD form and the 16 kD N-terminal cleavage product- the propiece of IL-1α, also known as the IL-1α N-terminal peptide (IL-1NTP). Intracellular ProIL-1α is present in many cells because they contain calpain inhibitors and are thus unable to process and secrete IL-1α (Afonina et al., 2011; Di Paolo and Shayakhmetov, 2013; Zheng et al., 2013). Recently, a novel mechanism to control IL-1α activity has been described by Zheng et al. (2013) and reviewed in Di Paolo and Shayakhmetov (2013). Thus, under normal conditions, IL-1α is synthesized as a p33 precursor that is sequestered in the cytosol by IL-1 receptor type 2 (IL-1R2), where it cannot be cleaved by proteases or activate IL-1 receptor type 1 (IL-1R1) signaling. However, under inflammatory signals, after inflammasome activation, IL-1R2 is cleaved by caspase-1 and ProIL-1α is further processed by calpain to the highly active p17 mature IL-1α form, which can be secreted from cells.

A biologically active membrane-associated form of IL-1α (23 kD) that is anchored to the membrane via a mannose-like receptor has been observed in activated cells that express the cytokine. However, it is not clear how IL-1α is inserted into the membrane.

IL-1α and IL-1β signal through the same IL-1Rs, which belong to the immunoglobulin (Ig) supergene family and are extensively expressed on many cell types. IL-1R1 (80 kD) is a signaling receptor, whereas IL-1R2 (68 kD) serves as a decoy target, acting to reduce excessive amounts of IL-1 (reviewed in Apte and Voronov, 2008; O'Neill, 2008; Dinarello, 2009, 2011b; Garlanda et al., 2009; Gabay et al., 2010; Sims and Smith, 2010; Voronov et al., 2013). Following the binding of IL-1 to IL-1R1, a second chain, i.e., the IL-1R acceptor protein (IL-1RAcP), is recruited. This heterodimeric complex triggers IL-1 signaling by activating the IL-1 receptor-associated kinase (IRAK) and ultimately leads to the activation of NF-κB and its target genes. On the contrary, IL-1R2 and the IL-1Ra do not form this heterodimeric complex with the IL-1RAcP and therefore do not recruit IRAK. While IL-1α and IL-1β signals through the same receptors, they differ dramatically in some biological functions (reviewed in Apte and Voronov, 2008; O'Neill, 2008; Dinarello, 2009, 2011b; Garlanda et al., 2009; Gabay et al., 2010; Sims and Smith, 2010; Voronov et al., 2013).

The differential effects of these molecules on angiogenesis are discussed in this review.

## The effects of IL-1 on ECs and its crosstalk with pro-angiogenic molecules

ECs are the main cells that are involved in both the normal and pathological angiogenic response. Multiple studies have shown *in vitro* effects of recombinant IL-1 on parameters related to the physiology of ECs, including their activation, as evidenced by morphological changes, increased migration and proliferation and ultimately organization into tube-like structures (reviewed in Voronov et al., 2007, 2010a, 2013; Apte and Voronov, 2008). As IL-1 is a strong activator of ECs, it induces profound changes in gene expression and function that allow these cells to participate actively in inflammatory reactions, immunity, and blood vessel formation. For example, IL-1β induces morphological transformation in human dermal micro-vascular endothelial cells (HDMECs), accompanied by an increased growth rate, loss of contact inhibition, and an increase in the permeability of confluent EC monolayers (Bokhari et al., 2006). Nevertheless, it is not known whether the described effects of IL-1 on ECs are direct, due to its action, or indirect, due to IL-1-induced cytokines produced by ECs. It was found that *in vitro*, IL-1β increases expression of FGF2 in ECs through activation of NF-κB (Lee and Kay, 2012) and also induces expression of different chemokines, cytokines, direct angiogenic factors, and adhesion molecules on ECs (Breviario et al., 1990; Sica et al., 1990; Kang et al., 2006). In addition, IL-1β interaction with IL-1R1 on ECs induces migration of the cells and tube formation, mainly via activation of p38-mitogen-activated protein kinase (MAPK) and MAPK-activated protein kinase 2 (Jagielska et al., 2012). Moreover, IL-1β up-regulates expression of VEGF and its receptors on ECs (Berse et al., 1999) or aortic smooth muscle cells (Stavri et al., 1995; Maruyama et al., 1999; Nasu et al., 2006) and VEGF secretion from these cells was significantly inhibited by the addition of IL-1Ra. *In vivo*, IL-1 also plays a synergistic, pro-angiogenic role with some pro-angiogenic factors (Friesel and Maciag, 1999). For example, the synergistic effects of VEGF and IL-1 in the up-regulation of genes of growth factors and inflammatory cytokines in ECs have been described and a 60% overlapping of genes induced by each of these cytokines was observed (Schweighofer et al., 2009). VEGF/IL-1 induced genes comprise mainly a group of genes with NFAT, as well as NF-κB binding sites in their promoters; VEGF-A preferentially uses NFAT and IL-1 uses NF-κB to induce these genes. It was also observed that both VEGF and IL-1β increase the permeability of ECs via a Src-dependent pathway (Sheikpranbabu et al., 2009).

Using a Matrigel model, we found that both rIL-1β and rVEGF induce each other and are both essential to induce an angiogenic response. Thus, neutralization of either of these cytokines abrogated the angiogenic response. The importance of the interaction between IL-1β and VEGF was also demonstrated by the weak angiogenic response observed in Matrigel plugs loaded with rVEGF in mice deficient in IL-1β or its signaling receptor (Carmi et al., 2013). Inhibition of VEGFR1 signaling in Matrigel plugs supplemented with recombinant cytokines (IL-1β or VEGF) abrogated angiogenesis, probably by inhibiting recruitment of bone marrow-derived (BMD) myeloid cells. Thus, in multiple studies, synergy of IL-1β and VEGF has been described; however, our studies demonstrate the necessity of both factors in angiogenic responses. IL-1β is also an essential factor for endothelial precursor cells (EPCs) to mature into ECs and this IL-1β-induced effect was facilitated by the addition of VEGF. For example, when either EPCs or myeloid circulating angiogenic cells from lupus patients were co-cultured with IL-1β and VEGF, their capacity to proliferate and differentiate into mature ECs was synergistically increased (Thacker et al., 2010).

There are fewer studies on the role of IL-1α in angiogenesis. We found that IL-1α has less potent pro-angiogenic effects compared to IL-1β in its recombinant form (unpublished results). Nevertheless, *in vivo* studies on the effects of IL-1α and IL-1β on angiogenesis revealed that both agonistic molecules are important, but probably work through different pathways. Thus, IL-1α can stimulate a high angiogenic response by recruiting macrophages that are an abundant source of FGF (reviewed in Sano et al., 1990; Brogi et al., 1993; Dinarello, 1996; Rider et al., 2011, 2012), or other VEGF-expressing inflammatory cells (Salven et al., 2002). In addition, a dose-dependent effect of IL-1α in *de novo* synthesis of VEGF by human peripheral mononuclear cells (PBMCs) was shown. This effect was blocked by treatment with VEGFR2 antibodies, whereas neutralization of VEGFR1 induced only marginal effects. Both *in vitro* and *in vivo*, IL-1α can stimulate ECs to secrete IL-8 and during *in vivo* angiogenesis, the source of IL-1 can be from PBMCs or activated platelets (Kaplanski et al., 1994a,b). IL-1α is also released from ECs following stress signals, such as starvation or TNF activation (Berda-Haddad et al., 2011) and in addition, IL-1α activates ECs to express CXCL1, VCAM-1, and ICAM-1, thus promoting trans-endothelial-migration of inflammatory cells (Thornton et al., 2010).

## IL-1 in inflammation-induced angiogenesis

In the last decade, the link between inflammation and angiogenesis has become recognized. Extensive angiogenesis was observed in chronic inflammation, for example in rheumatoid arthritis and inflammatory bowel disease (Folkman, 2001, 2003). In both acute and chronic inflammation, functional changes in the vasculature were found. In acute inflammation, these changes include an increase in permeability, extensive EC mitotic activity, and remodeling of capillaries. Upon chronic inflammation, vascular dilation and an increase in capillary density was observed. During inflammation, ECs actively recruit immune cells from the circulation into the underlying tissue, where they play a role in angiogenesis. An increase in growth factor and cytokine production, due to inflammation, also leads to proliferation of ECs.

The role of the IL-1 molecules in promoting inflammation-induced angiogenesis was studied by us, using the model of Matrigel plugs supplemented with supernatants of hypoxic macrophages, as such, or after activation with LPS as an inflammatory stimulus (Carmi et al., 2009). We showed that neutralization of IL-1 in supernatants of hypoxic macrophages, particularly IL-1β, completely abrogated cell infiltration and angiogenesis in Matrigel plugs, concomitant to dramatically reduced VEGF levels. Supernatants from macrophages of IL-1β knockout (KO) mice did not induce this inflammatory or angiogenic response. The importance of IL-1 signaling in the host was demonstrated by the dramatic reduction of inflammation-induced angiogenic responses in Matrigel plugs that contained supernatants derived from WT macrophages implanted in IL-1RI KO mice. Using the aortic sprouting assay, it was shown that IL-1 does not directly activate EC migration, proliferation, and organization into blood vessel-like structures, but rather activates infiltrating myeloid cells to produce a cascade of cytokines/chemokines, which further activate tissue resident ECs to produce direct pro-angiogenic factors, such as VEGF (Carmi et al., 2009). Similarly, Nakao et al., demonstrated that ectopic expression of IL-1β in the cornea induces increased angiogenesis, which is dependent on infiltration of COX-2-positive macrophages that activate the angiogenic process in a complex manner (Nakao et al., 2005). An example of an indirect effect of IL-1β on angiogenesis is the pro-angiogenic activity of osteopontin-treated monocytes on chick embryo chorioallantoic membranes that is completely abrogated by neutralization with anti-IL-1β antibodies (Naldini et al., 2006). In addition, systemic treatment with IL-1Ra prevented the formation of new blood vessels in corneas impregnated with VEGF or basic FGF (Coxon et al., 2002). In conclusion, IL-1, most probably the actively secreted form of IL-1β, functions directly or indirectly on myeloid infiltrating cells, as well as ECs, and thus regulates inflammation-induced angiogenic responses.

## IL-1 molecules play a key role in the tumor microenvironment

The induction of angiogenesis is considered to be a hallmark of cancer and is important for tumor growth and dissemination (Folkman, 1995, 2001, 2003; Folkman and D'Amore, 1996). An initially developing tumor is dormant until it undergoes the angiogenic switch, as initially described by Folkman. During this switch, the balance between the pro-angiogenic and anti-angiogenic factors changes and angiogenic stimuli are predominant. One of the major mechanisms of the angiogenic switch is enhanced expression and secretion of angiogenic factors, mainly VEGF, by the malignant cells. However, the mechanisms of the angiogenic switch are not completely elucidated, as detectable tumors usually have undergone this switch and already have an established vascular network. As indicated, tumor vascularization occurs through “classical” angiogenesis, as well as via vasculogenesis, which involves cells recruited from the BM (Carmeliet, 2005; De Palma et al., 2005; Bertolini et al., 2006). Thus, circulating VEGFR2-positive cells were shown to be initiators of vasculogenesis; however, conflicting findings on their ability to incorporate into newly formed blood vessels at tumor sites have been reported (De Palma et al., 2003; Gothert et al., 2004; Peters et al., 2005; Purhonen et al., 2008; Carmi et al., 2013). EPCs were initially described by Asahara, who reported on their ability to repair angiogenesis in ischemic tissues (Asahara et al., 1999). It was found that IL-1β can mobilize EPCs in a VEGF-dependent manner by regulation of VEGF and VEGFR2 expression on ECs, thus supporting neovascularization (Amano et al., 2004).

In further studies, a population of hematopoietic cells expressing endothelial markers, such as CD31, VEGFR2, and Tie2, was described and termed myeloid angiogenic cells (MACs) (Medina et al., 2010). These cells express high levels of pro-angiogenic and macrophage type 2 markers (Medina et al., 2011) and their function at the tumor site is still disputed. In some models, MACs were shown to differentiate into mature ECs that incorporate into the vasculature (Kawamoto et al., 2001; Urbich et al., 2003); however, upon intravitreal injection, MACs enhanced vascular repair of ischemic retinopathy, but preserved their original phenotype and did not differentiate into mature ECs. In addition, BMD cells of myeloid origin were shown to be essential for tumor-mediated angiogenesis. The proximity of BMD cells, including MACs, to ECs lining blood vessels suggests their paracrine role, facilitating the EC response during angiogenesis. These cells probably do not incorporate into blood vessels but function as supporting cells (Ziegelhoeffer et al., 2004).

Various populations of BMD myeloid cells are found in the tumor microenvironment. These include circulating monocytes, Tie-2 expressing monocytes (TEMs), myeloid-derived suppressor cells (MDSCs), tumor-associated macrophages (TAMs) and neutrophils (reviewed in Murdoch et al., 2008; Wels et al., 2008; Coffelt et al., 2010; Ferrara, 2010a; Ruffell et al., 2012; Sica et al., 2012; Chambers et al., 2013; De Palma and Lewis, 2013; Favre et al., 2013). The precise relationship among the myeloid cell types is uncertain and it is not known whether they act in the tumor microenvironment in redundant or specific manners. In various tumor experimental systems, diverse myeloid cell populations dominate. Mantovani et al. suggested a unifying hypothesis that myeloid cells at tumor sites actually represent a continuum of cell populations that all share properties of M2-macrophages, which promote tumor-mediated angiogenesis and progression (Mantovani and Sica, 2010; Allavena and Mantovani, 2012). Myeloid cells perform paracrine functions in the tumor-mediated environment, secreting angiogenic factors, and providing the inflammatory milieu for the angiogenic response. For example, MDSCs and TAMs secrete VEGF and MMP9, which increases the bioavailability of VEGF sequestered in the extracellular matrix by its proteolytic activity (reviewed in Murdoch et al., 2008; Mantovani et al., 2009; Gabrilovich et al., 2012).

IL-1, as a major hematopoietic and pro-inflammatory cytokine has dramatic effects on the generation of myeloid cells in the BM and on their recruitment to tumor sites, as well as on the *in situ* phenotype/function of these cells in the tumor microenvironment (reviewed in Apte and Voronov, 2008; Voronov et al., 2013). For example, *in vitro*, addition of IL-1 to BMCs increases secretion of VEGF and the total number of BMCs expressing CD34 or Flk-1 (Qin et al., 2006). Indeed, the absence of IL-1β in the tumor microenvironment limited the recruitment of FGF1-producing mononuclear cells to tumor sites (Prudovsky et al., 2003). Thus, it was suggested that IL-1 can affect BMD differentiation or induce them to secrete pro-inflammatory or angiogenic factors (reviewed in Apte and Voronov, 2008; Voronov et al., 2013), which are also involved in the recruitment of additional myeloid cells to tumor sites. Indeed, the importance of the VEGFR1/VEGF-A axis in recruiting myeloid cells that express VEGFR1 to tumor sites has been noted, and in mice deficient in VEGFR1, decreased recruitment of macrophages and other myeloid cells into tumors was observed (Duyndam et al., 2002). In addition, depletion of VEGFR1 in the renal cell carcinoma model leads to inhibition of macrophage recruitment to tumors (Li et al., 2011).

In our studies, the role of microenvironment-derived IL-1 on the recruitment of VEGFR1-positive BMD cells to tumor sites was confirmed. We characterized a new auto-induction circuit, IL-1β/VEGF, which acts via interactions between BMDs, mainly VEGFR1^+^/IL-1R1^+^ immature myeloid cells (MDSCs) and to a lesser extent macrophages, and tissue-resident ECs (Carmi et al., 2013). Myeloid cells do not directly stimulate ECs for migration, proliferation, and subsequent blood vessel formation. However, they produce IL-1β and a network of other pro-inflammatory cytokines/molecules, such as Bv8, CCL2 and CCL3, etc., which subsequently activate resting tissue-resident ECs to produce VEGF, as well as other direct pro-angiogenic factors, such as PlGF and bFGF. Thus, IL-1β provides the inflammatory microenvironment for angiogenesis and tumor progression. We have shown that IL-1β inhibition stably reduces tumor growth, by limiting inflammation and inducing the maturation of MDSCs into M1 macrophages, which do not promote tumor invasiveness and can be cytotoxic/cytostatic for tumor cells. In addition, M1 macrophages also serve as antigen-presenting cells for inducing anti-tumor immunity. These results suggest that IL-1β, apart from its ability to affect recruitment of myeloid cells, controls their maturation or activation for a pro-tumorigenic phenotype (Song et al., 2005; Bunt et al., 2006; Tu et al., 2008; Carmi et al., 2013). For example, Hagenmann et al. showed that IL-1R signaling in macrophages is essential for M2 polarization induced by conditioned medium of tumor cells (Hagemann et al., 2008). Other studies also showed the involvement of IL-1β in myeloid cell differentiation. Thus, IL-1β inhibits the skewing of myeloid cells present in proangiogenic cultures from circulating angiogenic cells to mature dendritic cells (DCs) (Mohty et al., 2003; Denny et al., 2007)**.** In addition, IL-1β can impair maturation in DCs treated with rapamycin (Turnquist et al., 2008).

Our experimental system of tumor cells encapsulated in Matrigel plugs allows the study of the early angiogenic response and even the angiogenic switch. In this case, VEGF produced by ECs possibly synergizes with direct angiogenic factors secreted by the malignant cells. Traditionally, it was thought that ECs respond to VEGF during the angiogenic response and produce very little or undetectable amounts of VEGF (Kerbel, 2008). In other studies, using large, established tumors that are densely infiltrated by “activated” myeloid cells, it was shown that VEGF mainly originates from such myeloid cells and it controls tumor angiogenesis (reviewed in Apte and Voronov, 2008; Murdoch et al., 2008; Coffelt et al., 2010; Ferrara, 2010a; Mantovani and Sica, 2010; Qian and Pollard, 2010; Ruffell et al., 2010; Allavena and Mantovani, 2012; Gabrilovich et al., 2012). In the early stages of tumor development, as assessed in our studies, myeloid cells are probably not yet sufficiently activated to produce VEGF. Thus, at different stages of tumor progression, VEGF is secreted by diverse microenvironmental cells and it acts together with angiogenic factors of tumor cell origin.

Results of our studies have shown that IL-1β is a major mediator in the tumor microenvironment, which plays a crucial role in the angiogenic response. IL-1β acts together with VEGF in mounting and maintaining tumor-mediated angiogenesis.

## The differential effects of IL-1 molecules in tumor-induced angiogenesis and invasiveness

High levels of IL-1 molecules are found in experimental tumor models and in human malignancies and IL-1 has been implicated as a key factor in tumor progression. IL-1 exerts its proliferative and angiogenic effects in the tumor microenvironment, mainly via interaction with stromal, inflammatory, as well as the malignant cells, stimulating tumor cell proliferation and invasion through autocrine or paracrine loops. For example, IL-1β induces secretion of chemokines by both tumor and tumor-microenvironmental cells and thus promotes cancer invasiveness (Portier et al., 1993; Suswam et al., 2005; Apte et al., 2006a; Naldini et al., 2010). A few studies have documented constitutive IL-1β protein production in human and animal cancer cell lines, including sarcomas and ovarian cell carcinomas (Dinarello, 1996; Lewis et al., 2006). In patients, overexpression of IL-1β has been described in solid tumors, including breast, colon, lung, head and neck cancers, and melanomas. In some tumors, IL-1, as well as other pro-inflammatory cytokines, is induced by oncogenes that transform the cell, thus providing the microenvironment for invasiveness of the malignant cells. In other types of tumors, IL-1 is induced only in the invasiveness and metastasis phases. The switch-on of IL-1 genes in malignant cells is induced by genetic alterations and possibly also by microenvironment-derived signals. Indeed, high IL-1 concentrations within the tumor microenvironment have been reported in numerous studies in cancer patients and experimental models and are associated with a more virulent tumor phenotype (reviewed in Elaraj et al., 2006; Lewis et al., 2006).

In the tumor milieu, IL-1 induces expression of various metastatic mediators, such as matrix metalloproteinases (MMP), VEGF, IL-8, IL-6, TNFα, and TGFβ (reviewed in Dinarello, 1996; Apte and Voronov, 2002, 2008; Apte et al., 2006b; Lewis et al., 2006; Voronov et al., 2013). Previously, in a transgenic model of Myc-dependent carcinogenesis, IL-1β was characterized as the principal effector molecule in the onset of angiogenesis, via MMP-mediated sequestration of extracellular matrix-associated VEGF and its further ligation to its cognate receptor on ECs (Shchors et al., 2006).

Our studies throughout the years have assessed the role of the IL-1 molecules in different phases of the malignant process, such as carcinogenesis, tumor angiogenesis, and invasiveness (Song et al., 2003, 2005; Krelin et al., 2007). Using a transplantable fibrosarcoma cell line transfected with cDNAs of the active forms of IL-1β, i.e., the mature form of IL-1β or the mature form of IL-1β ligated to a signal sequence (ssIL-1β), in which IL-1β is actively secreted through the endoplasmic reticulum-Golgi pathway, we observed that invasiveness of the different tumor cell lines was directly proportional to the amount of IL-1β secreted by the malignant cells (Song et al., 2003, 2005). In this system, invasiveness correlated with increased angiogenesis and accumulation of MDSCs in the spleen and tumor, which leads to general anergy. Similar observations were described in other experimental systems using IL-1β-transfected tumor cells (Saijo et al., 2002; Nakao et al., 2005; Bunt et al., 2006). We have hypothesized that initially small amounts of tumor cell-derived IL-1β induce a local inflammatory response, which subsequently recruits and activates BMDs that further secrete IL-1β, as well as an entire cytokine network that promotes tumor-mediated angiogenesis and tumor progression.

In various tumor models, we found that IL-1β induces increased tumor growth, invasiveness, and angiogenesis, when compared to IL-1α (Song et al., 2003; Voronov et al., 2003, 2010b). However, in some tumor cells, active secretion of IL-1α is observed (Apte and Voronov, 2008; Voronov et al., 2013).

In some tumors, such as breast carcinoma or pancreatic cancer, a pro-angiogenic signature correlated with IL-1α signaling (Matsuo et al., 2009; Guo and Gonzalez-Perez, 2011; Zhou et al., 2011). In these studies, IL-1α was shown to be involved in the induction of VEGF/VEGFR2 in the tumor microenvironment, through a cascade involving leptin/Notch. Secreted IL-1α may promote invasiveness in a similar manner to secreted IL-1β in these tumors.

However, in most malignancies, IL-1 is produced not only by tumor cells, but by other cells, such as neutrophils, macrophages, and MDSCs, recruited to the tumor microenvironment (reviewed in Apte and Voronov, 2008; Witz, 2008; Balkwill and Mantovani, 2010; Demaria et al., 2010; Grivennikov et al., 2010; Hanahan and Weinberg, 2011; Voronov et al., 2013).

These recruited cells thereby promote inflammation in the tumor microenvironment and thus increase the angiogenic response, tumor cell invasiveness and metastasis (Goldberg and Schwertfeger, 2010; Naldini et al., 2010; Schmid et al., 2011; Carmi et al., 2013).

Using IL-1 KO mice, we demonstrated that microenvironment-derived IL-1β, and to a much lesser extent IL-1α, is responsible for *in vivo* tumor angiogenesis and invasiveness of B16 melanoma cells. Significantly decreased angiogenesis was observed in IL-1β deficient mice injected with either melanoma cells or melanoma cell-containing Matrigel plugs, when compared to wild-type mice. In IL-1α KO mice, tumor growth and angiogenesis in Matrigel plugs were observed to a lesser extent than in WT mice, but higher than those in IL-1β KO mice (Voronov et al., 2003). Addition of recombinant IL-1 into Matrigel plugs containing B16 cells in IL-1β KO mice partially restored the angiogenic response, while addition of IL-1Ra to B16-containing Matrigel plugs in wild-type mice inhibited the ingrowth of the blood vessel network into the plugs. In subsequent studies, the angiogenic potential of IL-1 was confirmed by Elaraj et al., who observed high expression of IL-1 mRNA in more than half of all tested metastatic human tumor specimens, including non-small-cell lung carcinoma, colorectal adenocarcinoma, and melanoma tumor samples. Supernatants from IL-1 expressing cell lines induced a significant increase in EC monolayer permeability, a hallmark event in early angiogenesis. Systemic treatment with recombinant IL-1Ra resulted in significant inhibition of xenograft growth and neovessel density of IL-1-producing tumor cell lines. Nevertheless, the addition of IL-1Ra did not have any effect in non-IL-1-producing tumor lines (Elaraj et al., 2006).

## Anti-IL-1 therapy in anti-tumor approaches

Since tumor development is related to and dependent on the angiogenic response, anti-angiogenic therapy targeting blood vessel formation was used to suppress existing tumors and to prevent metastasis. Various studies used different anti-angiogenic molecules or agents to limit tumor angiogenesis. The main molecule used in these experiments was VEGF, which is the most abundant pro-angiogenic molecule. A number of preclinical studies demonstrated a significant inhibition of tumor growth in various types of cancer by blocking VEGF (Ferrara and Kerbel, 2005). VEGF has been approved by the FDA to be used in cancer patients to inhibit tumor growth. Anti-VEGF monoclonal antibodies (bevacizumab, also called Avastin) were tested in various metastatic cancers in humans, usually in combination with chemotherapy (Ferrara et al., 2005; Carmeliet and Jain, 2011). However, some patients are refractory to this treatment and tumor recurrence or even metastases were observed after initial tumor shrinkage. It is beyond the scope of this review to summarize the effects of anti-VEGF therapy in cancer patients. In animal models, it was also shown that termination or interruption of VEGF neutralization induces rapid vascular regrowth in tumors and limits tumor-suppressive effects of VEGF treatment (reviewed in Kubota, 2012; Bellou et al., 2013). The dramatic response of the tumor microenvironment to VEGF inhibition may be due to the key role VEGF plays in normal cell viability, including ECs, myeloid cells, and possibly other cell types involved in tissue homeostasis (Chung and Ferrara, 2011; Lazarus and Keshet, 2011; Luo et al., 2011; Potente et al., 2011). For example, blocking VEGF induces EC apoptosis, resulting in the loss of integrity of blood vessels, and disturbance of tissue homeostasis. Due to these effects, VEGF blockage damages not only tumor blood vessels, but also healthy vessels, thus occasionally resulting in severe complications, such as hemorrhagic or thrombotic events (Verheul and Pinedo, 2007).

Different mechanisms have been proposed to explain rebound angiogenesis following VEGF neutralization (Casanovas et al., 2005; Ferrara, 2010b; Chung and Ferrara, 2011; Carmi et al., 2013). For example, intrinsic mechanisms, such as the adaptation of malignant cells to low VEGF levels by selecting tumor cell variants that are less dependent on VEGF for their survival/proliferation or variants with enhanced expression/secretion of other pro-angiogenic factors, such as bFGF or PlGF have been described (Casanovas et al., 2005; Fischer et al., 2007; Kubota, 2012). Following VEGF neutralization, extrinsic mechanisms also contribute to tumor recurrence by altering microenvironment cells to express different or additional angiogenic factors, thus facilitating tumor recurrence. These include induced secretion of Bv8 by MDSCs and a subsequent VEGF-independent angiogenic response (Shojaei et al., 2007; Carmi et al., 2013) or expression of PDGF-C in cancer-associated fibroblasts (Bergers and Hanahan, 2008; Crawford et al., 2009). In our studies, we described a novel mechanism of rebound angiogenesis after VEGF inhibition in Matrigel plugs containing tumor cells (Carmi et al., 2013). This resulted in significant and consistently elevated expression of direct angiogenic factors, such as bFGF, PlGF, and PDGF, as well as VEGF in VEGFR1^+^ immature myeloid cells, mostly MDSCs. The re-programming of VEGFR1^+^ immature myeloid cells into active pro-angiogenic cells also involves increased expression of hypoxia-inducible factor 1α, a key transcription factor regulating angiogenesis (Cramer and Johnson, 2003; Semenza, 2011). In contrast, in “uninterrupted angiogenesis” (without anti-VEGF treatment), as described above, ECs, rather than myeloid cells, are the major cells that produce VEGF and other direct angiogenic factors (Carmi et al., 2013).

In recent years, myeloid, cell-driven angiogenesis was shown to contribute to refractoriness or resistance to VEGF inhibition. For example, as discussed above, MDSCs can secrete Bv8, which further upregulates granulocyte colony-stimulating factor that promotes tumor growth and angiogenesis in a VEGF-independent manner. Therefore, intervention in myeloid cell recruitment to tumor sites in order to modulate differentiation or neutralization of specific pro-inflammatory or pro-angiogenic factors secreted by these cells, are now widely discussed. For example, blocking CSF-1 was shown to be effective in suppressing tumor angiogenesis (Kubota, 2012). In our studies, we found that blocking a major, alarm, pro-inflammatory molecule derived from myeloid cells, i.e., IL-1β can lead to a significant amelioration of tumor angiogenesis (Voronov et al., 2003; Carmi et al., 2013). The inhibition of IL-1β does not affect the healthy vascular system or tissue homeostasis and is more suitable for anti-angiogenic therapy than anti-VEGF approaches. IL-1β neutralization largely inhibited infiltration of myeloid cells, which are obligatory for tumor-mediated angiogenesis. Furthermore, IL-1β neutralization induces maturation of immature myeloid cells into anti-invasive M1 macrophages.

We recently found that IL-1β neutralization inhibited pro-inflammatory genes, such as Bv8, CCL2 and CCL3 in myeloid cells, and did not significantly alter the expression of angiogenic factors. IL-1β inhibition also reduced *in vivo* tumor development in mice injected subcutaneously with tumor cells. This inhibition was long-lasting and without recurrence a contrast to that observed with anti-VEGF (Carmi et al., 2013).

These results have provided the rationale to use IL-1β neutralization as a treatment for tumor-mediated angiogenesis, and subsequent tumor progression.

Indeed, the positive role IL-1Ra plays in reducing the angiogenic response and in attenuating tumor progression has been demonstrated by us (Song et al., 2003; Voronov et al., 2003) and by others (Saijo et al., 2002; Nakao et al., 2005; Bunt et al., 2006; Schmid et al., 2011). IL-1 neutralization agents are available at the present time (Dinarello, 2010a,b; Dinarello and van der Meer, 2012). The IL-1Ra, also called Anakirna (Kineret; Amgen/Biovitrum) has been shown to be safe and effective in alleviating symptoms of rheumatoid arthritis and other auto-inflammatory diseases. Continuous delivery systems of recombinant IL-1Ra or cells over-expressing IL-1Ra, encapsulated within alginate-poly (L-lysine)-alginate (APA) microspheres, reduced the tumor burden and inhibited tumor-mediated angiogenesis when implanted into tumor-bearing mice (Bar et al., 2004; Lavi et al., 2007). Similarly, over-expression of the IL-1Ra in human melanoma cell lines expressing endogenous IL-1, inhibits tumor growth and metastasis in human melanoma xenografts in nude mice through its effects on the microenvironment (Weinreich et al., 2003; Elaraj et al., 2006). Altogether, these results provide pre-clinical support for the use of IL-1Ra in cancer therapy.

Recently, some novel IL-1 neutralization agents, such as humanized anti-IL-1β, an IL-1 trap, as well as other agents that indirectly inhibit IL-1β production, have been developed (Dinarello, 2011b). These agents are now ready for testing in cancer patients. Optimally, IL-1 neutralization should be most effective in patients with minimal residual disease (MRD), to prevent tumor-induced angiogenesis, recurrence, and metastasis.

## Conclusions

**Figure 1:**
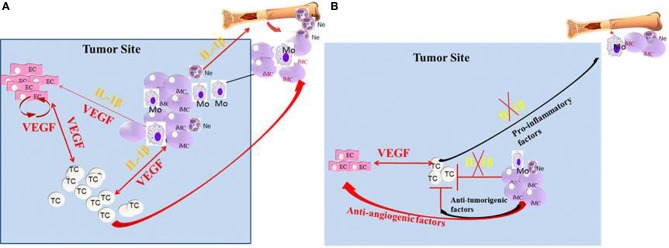
**Crosstalk between IL-1β and VEGF in the tumor microenvironment. (A)** In the tumor microenvironment, IL-1β can be expressed initially by the malignant cells. In turn, IL-1β can recruit myeloid cells from the BM and further activate them to secrete pro-inflammatory and pro-angiogenic molecules, such as VEGF and IL-1β. Furthermore, IL-1β keeps myeloid cells in their immature stage, where they are pro-invasive and immunosuppressive. IL-1β can also activate tissue-resident ECs to produce VEGF and other pro-angiogenic factors. The malignant cells can also be activated by IL-1β for increased invasiveness. VEGF in the tumor microenvironment activates ECs and myeloid cells and also has homeostatic effects on these cells. **(B)** In the absence of IL-1β, inflammation, as well as VEGF production is diminished, which results in reduced invasiveness. Lack of IL-1β in the tumor microenvironment also induces the maturation of MDSCs into anti-tumor M1 macrophages.

Finally, this review demonstrated the important role of the IL-1 family molecules, especially IL-1β, in the tumor microenvironment. IL-1β represents a major upstream cytokine that controls the local pro-inflammatory cascade and thereby affects the balance between protective immunity and destructive inflammation. IL-1 modulates diverse cells in the tumor microenvironment and acts together with VEGF in mounting and maintaining tumor-mediated angiogenesis (Figure 1). Thus, its neutralization should reduce tumor progression and invasiveness via its affect on pro-tumorigenic cells, and on the tumor-induced angiogenic switch. Further characterization of the optimal conditions for IL-1 neutralization could lead to the application of anti-IL-1 approaches in cancer therapy.

### Conflict of interest statement

The authors declare that the research was conducted in the absence of any commercial or financial relationships that could be construed as a potential conflict of interest.

## Abbreviations

IL-1: Interleukin-1
IL-1Ra: Interleukin-1 receptor antagonist
VEGF: Vascular endothelial growth factor
VEGFR: Vascular endothelial growth factor receptor
TNF: Tumor necrosis factor
TGFβ: Tumor growth factor beta
PlGF: Placental growth factor
bFGF: Basic Fibroblast growth factor.
